# Assessment of Blood Glucose and Electrolytes during Cardiopulmonary Bypass in Diabetic and Non-Diabetic Patients of Pakistan

**DOI:** 10.5539/gjhs.v8n9p159

**Published:** 2015-01-22

**Authors:** Muhammad Bilal, Abdul Haseeb, Mohammad Hassaan Khan, Akash Khetpal, Muhammad Saad, Mohammad Hussham Arshad, Mudassir Iqbal Dar, Najya Hasan, Rafia Rafiq, Maryam Sherwani, Haider Abbas, Ayesha Sultan, Maha Inam

**Affiliations:** 1 Dow University of Health Sciences, Karachi, Pakistan; 2 Aga Khan University Hospital, Karachi, Pakistan; 3 South City Hospital, Karachi, Pakistan; 4 Cardiac Surgery Department, Civil Hospital, Karachi, Pakistan; 5 Department of Biological Sciences, Cedar College, Karachi, Pakistan; 6 Department of Biological Sciences, Karachi Grammar School, Karachi, Pakistan

**Keywords:** coronary artery bypass grafting surgery, cardiopulmonary bypass, diabetic patient, hyperglycemia, electrolytes, CPB: Cardiopulmonary Bypass, CABG: Coronary Artery Bypass Grafting, FFA: Free Fatty Acid, LVEF: Left Ventricular Ejection Fraction, ANOVA: Repeated-Measures Analysis Of Variance

## Abstract

**Introduction:**

Perioperative hyperglycemia has been shown to be related to higher levels of morbidity and mortality in patients on cardiopulmonary bypass (CPB) undergoing coronary artery bypass grafting (CABG), both diabetic and non-diabetic. Blood electrolytes, like sodium, potassium, calcium, and chloride play a very important role in the normal functioning of the body and can lead to a variety of clinical disorders if they become deficient. A minimal number of studies have been conducted on the simultaneous perioperative changes in both blood glucose and electrolyte levels during CPB in Pakistan. Therefore, our aim is to record and compare the changes in blood glucose and electrolyte levels during CPB in diabetic and non-diabetic patients.

**Materials and Methods:**

This was a prospective, observational study conducted on 200 patients who underwent CABG with CPB, from October 2014 to March 2015. The patients were recruited from the Cardiac Surgery Ward, Civil Hospital Karachi after they complied with the inclusion criteria. Repeated-measures analysis of variance (ANOVA) was used to compare the trend of the changes perioperatively for the two groups.

**Results:**

There was no significant difference in changes in blood glucose between the two groups (P = 0.62). The only significant difference detected between the two groups was for PaCO_2_ (P = 0.001). Besides, further analysis revealed insignificant group differences for the trend changes in other blood electrolytes (P > 0.05).

**Conclusion:**

Our findings highlighted that there is no significant difference in blood electrolytes changes and the increase in blood glucose levels between diabetic and non-diabetic patients.

## 1. Introduction

Perioperative hyperglycemia has been shown to be related to higher levels of morbidity and mortality in patients on cardiopulmonary bypass (CPB) undergoing coronary artery bypass grafting (CABG), both diabetic and non-diabetic (Estrada, Young, Nifong, & Chitwood, 2003). While undergoing surgery, the surgical stress and the form of anesthesia given lead to the increased release of stress hormones, like catecholamines, cortisol, glucagon and growth hormone, which disturb glucose homeostasis by inducing a catabolic state with protein and fat mobilization, leading to increased blood glucose levels (Ellger et al., 2006). Additionally, postoperative hyperglycemia is also seen in patients due to an increase in insulin resistance and its decreased secretion caused by surgical trauma, a phenomenon called ‘surgical/stress diabetes’ or ‘diabetes of injury’ (Vanhorebeek, & Langouche, 2009). It is also claimed that increased lipolysis due to the stress hormones results in increased free fatty acid (FFA) levels that produce insulin-resistance in diabetic and non-diabetic patients, further contributing to stress-related hyperglycemia (Roden et al., 1996). There is a greater chance of severe hypoglycemic episodes in patients who are obese, hypertensive and have an atherogenic lipid profile. These characteristics are very common in cardiac surgery patients (Weintraub, Wenger, Jones, Craver & Guyton, 1993). Consequently, prolonged hyperglycemia is associated with many adverse changes in the body, like promoting pro-inflammatory and pro-coagulatory environments (Lazzeri, Tarquini, Giunta, & Gensini, 2009). It also affects the extent of ischemic damage to the heart as it increases the severity of reperfusion injuries and initiates coronary endothelial cell dysfunction (Gross et al., 2003).

Blood electrolytes, like sodium, potassium, calciumand chloride play a very important role in the normal functioning of the body (Ducceschi et al., 2000). If they are deficient, they can lead to a variety of clinical disorders, especially arrhythmias in cardiac surgery patients (Webster, Brady, & Morris, 2002). Moreover, hypokalemia has been clearly shown to be associated with increased risk of hyperglycemia (Polderman, & Girbes, 2004). Post-operative atrial fibrillation (after CABG) was connected to increased chloride and decreased phosphate levels immediately post-surgery (Svagzdiene, & Sirvinskas, 2006). These adverse effects can be prevented therefore blood electrolyte levels should be routinely checked perioperatively, especially in patients undergoing cardiac surgery.

There are many studies that have individually compared either hyperglycemia or serum electrolyte levels with their outcomes in cardiac surgery patients. However, according to our knowledge, a minimal number have been carried out in Pakistan. Also, very few studies have also been conducted on the simultaneous perioperative changes in both blood glucose and electrolyte levels during CPB. It is anticipated that changes in blood glucose and electrolyte balance may contribute some morbidity in patients under CPB, therefore, our aim is to record and compare the changes in blood glucose and electrolyte levels during CPB in diabetic and non-diabetic patients undergoing CABG surgery in Pakistan.

## 2. Methodology

This prospective, observational study was conducted on 200 patients who underwent CABG with CPB, from October 2014 to March 2015. The patients were divided into two equal groups of 100- diabetic and non-diabetic. The subjects selected were those who were admitted to the Cardiac Surgery Ward, Civil Hospital Karachi, Pakistan and were aged 30 to 65 years old. The exclusion criteria was such that patients with chronic underlying illnesses, like liver or kidney problems, patients operated on valvular surgeries along with CABG, those weighing over 110 kg and participants having an HbA1c over 7.5 were not included in the study. This study was approved by the review board of Dow University of Health Sciences. All other ethical requirements including informed consent and confidentiality of participant’sdata were ensured.

The methodology that was used by Maasoumi et al in a previous study conducted at Rajaei Cardiovascular Medical and Research Center, Tehran, Iran was followed (Maasoumi, & Saberi, 2013). According to that, patients received a continuous dose of regular insulin during 24 hours prior to the surgery. Regular insulin was also administered intra-operatively if blood glucose was over 200 mg/dL as suggested by Blaha et al (2009). The dosages varied accordingly: 4 U/kg for blood glucose of 200-250; 8 U/kg for blood glucose of 251-300; 12 U/kg for blood glucose of 301-350.

All the patients received the same medications perioperatively according to the protocol. 1 mg oral lorazepam was given, once on the night before the operation and once at the morning of the operation day, while 0.1 mg/kg morphine was given an hour pre-operatively. The anesthesia was also performed identically, with the same technician catering to all the patients. 1 mg/kg midazolam, 0.5 µg/kg sufentanil, 1-2 mg/kg propofol, and 0.2 mg/kg cisatracurium along with 500 ml ringer solution were used for the anesthetic approach. It was maintained using 20 - 100 µg/kg/min propofol, 0.25 - 0.5 µg/kg/min midazolam, fentanyl 0.5 µg/kg/hour, and 2-4 µg/kg/min atracurium in accordance with the methodology of Golamreza Maasoumi et al. (Maasoumi, & Saberi, 2013).

A cannula was inserted in the left radial artery to acquire blood samples. The levels of bicarbonate (HCO_3_), sodium (Na), potassium (K), chloride (Cl), calcium (Ca), glucose, lactate, pH and pCO_2_ in blood were measured at three points: pre-operatively after the insertion of the cannula and before the beginning of anesthesia induction; intra-operatively during CPB; and post-operatively 10 to 15 minutes after termination of CPB. To increase the accuracy of measurement intra-operatively, three readings were taken during CPB (two before and during cold phase, and one after warm phase) and the mean value was calculated and recorded.

The methodology of our study was pre-tested on 30 participants in the same setting and collected data was analyzed to evaluate the feasibility of the study and to remove the ambiguities in the methodology before its implementation. The data from preliminary trail was not included in the full-scale research project.

The data was entered on Social Sciences (SPSS) software program for Windows, version 19.0 and the same software was used for data management and analysis. Moreover, we used repeated-measures analysis of variance (ANOVA) to assess and to compare the changes occurring for different variables before, during and after CPB for the two groups. A *P* value less than 0.05 was considered to be statistically significant.

## 3. Results

A total of 100 diabetic (42 males and 58 females) and 100 non diabetic (49 males and 51 females) patients, scheduled for an elective CABG surgery on CBP were included in the study. The sex differences were statistically insignificant (*P* = 0.345). The mean age for the diabetic patients was 56.20±8.23 while non diabetic patients aged 60.13±6.10. Moreover, the HbA1c of the diabetics ranged from 5.0% to 6.9%, which indicated a good control over their blood glucose level in last 3 months.

**Figure 1:**
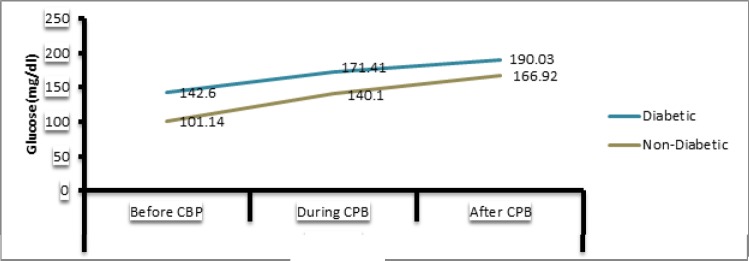
Illustrates trend changes in blood glucose level among booth the groups perioperatively

Furthermore, blood glucose and electrolytes were statistically analyzed before, during and after CPB in the both groups as shown in Table 1. The blood glucose level changed from the baseline of 143 to 171 in diabetics and rose from 101 to 140 in non diabetics during CPB. However, this change is statistically insignificant as indicated by p value of 0.62. Apart from glucose levels, PaCO_2_ was almost same for both the groups (31 mmHg) at the baseline level. The repeated-measures ANOVA indicated a significant change in non diabetics during operation (p value=0.001). Furthermore, Lactate used as an indicator of tissue perfusion, changed insignificantly in both the groups during and after CPB (p>0.05). Both the diabetics and non diabetics needed the same amount of Ca during CPB. Besides, further analysis revealed insignificant group differences for other blood electrolytes (*P* > 0.05). Figure 1 illustrates trend changes in blood glucose level among booth the groups perioperatively.

**Table 1:** Depicts blood glucose and electrolytes in diabetic and non-diabetic patients

Parameter	Diabetic (mean±SD)			Non-Diabetic (mean±SD)			P-value		

	Before CBP	During CPB	After CPB	Before CPB	During CPB	After CPB	Before CPB	After CPB	Repeated measures ANOVA
Glucose(mg/dl)	142.60±51.03	171.41±36.18	190.03±54.20	101.14±15.89	140.10±28.78	166.92±36.10	0.001	0.05	0.62
Lactate(meq/l)	1.17±0.38	2.13±0.66	2.81±0.93	1.19±0.56	2.37±0.87	2.63±1.01	0.501	0.77	0.30
PaCO_2_(mmHg)	30.87±5.11	32.11±7.55	31.68±5.40	30.63±6.23	36.60±5.11	34.99±6.91	0.301	0.01	0.001
HCO_3_(meq/l)	21.91±1.88	20.40±2.65	19.75±2.61	21.33±3.56	20.51±2.39	19.49±2.39	0.200	0.80	0.29
pH	7.46±0.06	7.20±0.04	7.37±0.05	7.43±0.05	7.41±0.09	7.37±0.5	0.638	0.29	0.08
Na(meq/l)	140.98±5.17	133.99±3.98	138.65±3.71	140.99±4.16	134.98±3.72	138.62±4.60	0.599	0.87	0.88
K (meq/l)	3.39±0.38	4.60±0.79	4.07±0.61	3.39±0.35	5.21±0.75	4.35±0.69	0.412	0.33	0.60
Cl(meq/l)	105.20±4.69	110.30±4.30	107.99±4.10	105.78±5.33	113.13±14.0	107.38±5.89	0.629	0.31	0.19
Ca(meq/l)	1.81±0.33	2.15±0.29	1.87±0.49	1.59±0.27	2.31±0.36	1.79 ±.40	0.071	0.66	0.13

## 4. Discussion

In general, our results indicate that blood glucose levels rose significantly during and after the surgery for both the groups and the trend remained consistent throughout. Apart from that, there was no significant change in the level of blood electrolytes perioperatively. Moreover, significant fluctuations were observed in the levels of PaCO_2_ for both diabetics and non-diabetics. These results are congruent with the findings of Maasoumi et al. (Maasoumi, & Saberi, 2013).

Since CPB is considered a major causative agent for hyperglycemia intra- and post-operatively, the study was conducted to investigate the alteration in blood glucose levels during CPB in CABG patients (Rassias & Yeager, 2003). In our diabetic patients, the blood glucose value rose from 143 mg/dL to 171 mg/dL while the increase was from 101 mg/dL to 140 mg/dL in non-diabetic patients. These results reiterate the fact that glucose tolerance persists in patients regardless of their being diabetic or not which is in line with the findings of Thorell et al. (Thorell, Nygren, & Ljungqvist, 1999). Furthermore, our results endorse the views of previous studies that CPB disturbs glucose homeostasis (Knapik et al., 2009). The continuous insulin infusion during CPB should ideally have prevented high blood glucose levels, however, the release of stress hormones due to surgical stress resulted in insulin resistance, leading to prolonged hyperglycemia (Lehot, Piriz, Villard, Cohen & Guidollet, 1992). The other contributing factors include hypothermia (Anderson, Brismar, Barr, & Ivert, 2005), hyperthermia (Anderson et al., 2005) and non-esterified fatty acid secretion due to heparin infusion during CPB (Van den Berghe et al., 2003).

We anticipated that the blood glucose would increase at a greater rate in diabetic patients as compared to non-diabetic. This is due to the fact that glucose homeostasis is greatly altered in the former. However, despite having higher blood glucose levels in diabetic patients, the trend remained similar throughout as indicated by repeated me (Polderman, & Girbes, 2004) asure ANOVA. This could be attributed by assuming that both groups undergo insulin resistant due to surgical stress. Prompt treatment of elevated post operative blood glucose level is essential as previous studies conducted by Van den Berghe and associates (Furnary et al., 2003), publications by the Furnary group (McAlister, Man, Bistritz, Amad & Tandon, 2003), and manyothers have indicated high morbidity and mortality in both the groups.

Previous studies have revealed that electrolyte consumption increases during CPB due to intracellular shift and increased urinary excretion (Polderman, Bloemers, Peerdeman, & Girbes, 2000). However, our study showed an insignificant decrease in electrolyte levels, with the levels remaining roughly constant during the procedure. These findings are inconsistent with the work of Polderman et al who found that significant electrolyte depletion occurs in the rewarming phase due to hypothermia induction (Polderman et al., 2000). Our findings could be explained owing to the fact that all patients received potassium supplementation throughout the surgery in addition to potassium rich cardioplegia solution.

Moreover, our results revealed a statistically insignificant (*P* = 0.432) rise in PaCO_2_ among non diabetic patients. Similarly, an insignificant difference was also observed for group stratification among diabetic participants (*P* = 0.124). Conversely, a significant difference was observed in trend changes between diabetics and non diabetics. These findings are inconsistent with work done by Chaney et al. (Chaney, Nikolov, Blakeman, & Bakhos, 2000) and Loer et al. (Loer, Kalweit, & Tarnow, 2000). The differences could be linked to our limited sample population.

To the best of our knowledge, this is the pioneer study relating trend changes of blood glucose levels and electrolytes among diabetic and non diabetic patients undergoing CABG on CPB in Pakistan. However, we encountered some limitations including limited sample size and unavailability of a comparison group of patients undergoing CABG without CPB. Therefore, further researches need to be carried out on glycemic control and electrolyte changes for cardiac surgery patients, with or without CPB, in order to increase the level of healthcare for these patients.

## 5. Conclusion

In summary, our findings highlighted that there is no significant difference in blood electrolytes changes and the increase in blood glucose levels between diabetic and non-diabetic patients.
